# A Magnetorheological Fluids-Based Robot-Assisted Catheter/Guidewire Surgery System for Endovascular Catheterization

**DOI:** 10.3390/mi12060640

**Published:** 2021-05-30

**Authors:** Linshuai Zhang, Shuoxin Gu, Shuxiang Guo, Takashi Tamiya

**Affiliations:** 1 School of Control Engineering, Chengdu University of Information Technology, Chengdu 610225, China; linshuai@cuit.edu.cn; 2 Faculty of Engineering and Design, Kagawa University, Takamatsu 761-0396, Japan; 3 Key Laboratory of Convergence Medical Engineering System and Healthcare Technology, the Ministry of Industry Information Technology, School of Life Science, Beijing Institute of Technology, Beijing 100081, China; 4 Department of Neurological Surgery, Faculty of Medicine, Kagawa University, Takamatsu 761-0396, Japan; tamiya@kms.ac.jp

**Keywords:** robot-assisted catheter operating system, magnetorheological fluids, endovascular catheterization, collision detection, vascular interventional surgery

## Abstract

A teleoperated robotic catheter operating system is a solution to avoid occupational hazards caused by repeated exposure radiation of the surgeon to X-ray during the endovascular procedures. However, inadequate force feedback and collision detection while teleoperating surgical tools elevate the risk of endovascular procedures. Moreover, surgeons cannot control the force of the catheter/guidewire within a proper range, and thus the risk of blood vessel damage will increase. In this paper, a magnetorheological fluid (MR)-based robot-assisted catheter/guidewire surgery system has been developed, which uses the surgeon’s natural manipulation skills acquired through experience and uses haptic cues to generate collision detection to ensure surgical safety. We present tests for the performance evaluation regarding the teleoperation, the force measurement, and the collision detection with haptic cues. Results show that the system can track the desired position of the surgical tool and detect the relevant force event at the catheter. In addition, this method can more readily enable surgeons to distinguish whether the proximal force exceeds or meets the safety threshold of blood vessels.

## 1. Introduction

A report from the American Heart Association (AHA) shows that cardiovascular and cerebrovascular diseases have become one of the three leading causes (heart disease, stroke, and vascular diseases) of death in humans. Even in developed countries, the number of deaths from cardiovascular diseases is still up to 34% each year, posing a serious threat to human health [1]. With the rapid development of medical technology, vascular interventional surgery (VIS) has attracted wide attention because it has the advantages of a small incision, rapid recovery, and fewer complications. It has become the common practice for diagnosing and treating various cardiac and vascular diseases, such as arterial stenosis, thrombosis, and atherosclerosis. During the endovascular procedures, a flexible catheter and guidewire that is usually inserted along the patient’s blood vessels through a small incision of the femoral artery in the groin or the radial artery in the wrist into the target of the lesion. This procedure usually uses a digital subtraction angiography (DSA) system to visually assist the interventionist during intravascular navigation and eventual catheter placement [2,3]. Nonetheless, the success of the surgery will be affected by the surgeon’s fatigue and physical tremors, and a risk to the surgeon’s health will be posed due to prolonged radiation exposure [4,5]. Moreover, an interventional surgery involves high risks, and the surgeon must be highly skilled and professional.

### 1.1. Related Work

In recent years, robot-assisted technology has played an important role in the field of medicine. To address the above shortcomings, researchers combine robot-assisted technology with VIS to develop many kinds of robot-assisted catheter operating systems for endovascular procedures. Among commercialized robot-assisted catheter operating systems, the Sensei X robotic catheter system (Hansen Medical, Inc., Mountain View, CA, USA) is a flexible robotic platform that combines advanced 3D catheter control technology with 3D visualization. It is a combination of collaborative technologies that provide physicians with greater accuracy and stability [6,7]. The Corpath robotic system (Corindus, Inc., Natick, MA, USA) allows the interventionist to perform percutaneous coronary intervention (PCI) procedures remotely from the console. The robot-assisted system can synchronously operate a catheter and a guidewire during intravascular surgery [8,9]. The Amigo TM (Catheter Robotics, Inc., Mount Olive, NJ, USA) remote catheter system (RCS) can safely and effectively insert a catheter into the right side of the heart [10].

Numerous other devices have also been developed by researchers around the world, and they have made significant contributions to improving techniques for intravascular surgeries. A novel remote catheter surgery system has been developed by Tavallaei and colleagues that can reduce the operator’s physical stress and radiation during two-dimensional X-ray image guidance. This system is compatible with magnetic resonance imaging (MRI) to perform catheterization due to the use of ultrasonic motors in the catheter manipulator (slave) [11]. He has also developed a new compact three-degree-of-freedom remote-controlled robotic catheter system that enables interventional surgeons to skillfully guide conventional catheters in a safe location. The system evaluation was performed ex vivo, and the safety of the system was demonstrated by the in vivo experiments [12]. More recently, an assembly-type and low-cost human-machine interaction catheter driving system with four DoFs was proposed to reduce radiation exposure. The performance of bilateral control was evaluated by the optical tracking experiments [3]. These robotic systems can drastically reduce radiation exposure and improve the stability of catheter operation by replacing human effort for high radiation exposure procedures.

However, surgeons face several challenges to advance the catheter in the complicated and delicate vascular structure of human beings, especially under fragile and tortuous blood vessels. It has been reported that the tortuosity of vessels is one of the main failure reasons in endovascular procedures [13]. Surgeons usually rely on visualization to avoid major collisions of vessels during catheterization. It is difficult to determine whether vascular collisions have occurred due to the lack of depth perception and low-resolution perspective images [14]. Thus, some researchers have built force-measuring devices into robotic catheter systems to display forces in blood vessels more intuitively during the endovascular procedure. A master-slave catheter driving system was designed to operate a steerable catheter with positioning function under the 3D guiding image, and four force sensors were placed into the insertion mechanism to realize force measurement [15]. Guo et al. proposed a novel master-slave robotic catheter operating system with force feedback and visual feedback to train the novices to perform vascular interventional surgery [16]. Bao et al. proposed a remote-controlled vascular interventional robot based on an electromagnetic clutch to perform the catheter insertion with force feedback [17]. In addition, two identical ones are connected in series from the manipulator to achieve coordinated movement of the guidewire and catheter. The system was evaluated by human experiments [18]. Based on the application of a force sensor, Guo et al. adopted the closed-loop force feedback in the interventional surgical robotic system to provide adequate haptic feedback and improve control accuracy during catheterization [5]. To improve the accuracy of force measurement, the compact and economical force-sensing device based on strain gauges was directly connected to the catheter gripper so as to avoid measurement errors caused by mechanical transmission [19,20]. To reduce the collision trauma during the catheterization, Yin et al. designed a haptic interface to detect the collision between the tip of a catheter and the blood vessel during surgery [21,22]. On this basis, Wang et al. introduced a speed-adjustable mechanism (SAM) installed in front of the haptic interface device for tissue protection [23]. A robot-assisted collision protection mechanism for vascular interventional surgery based on passive compliance control was presented, and its feasibility was verified by relevant control algorithms and a series of relevant experiments [24]. Nevertheless, for the majority of the systems in this field, the sensors either mounted on the robot or the surgical device have been used to capture tool-tissue interaction force, and surgeons must monitor the trends and values of forces in real-time to determine whether a collision has occurred. Keeping focused at all times makes operators more likely to be fatigued. In addition, for most systems, human-robot inputs typically use a joystick or haptic interface with multiple degrees of freedom, thereby eliminating traditional catheterization techniques in actual surgeries. Moreover, surgeons cannot control the force of catheter/guidewire within a proper range, and thus the blood vessel damage will increase for certain. Motivated by such consideration, it is necessary to develop teleoperated robotic catheter operating systems with more ergonomic interfaces that exploit the natural manipulation skills of surgeons, while in some instances, they also augment the surgeon’s ability to conduct the procedure by providing collision detection with haptic cues. This method can enable surgeons to distinguish whether the proximal force exceeds the safety threshold of blood vessels or not more easily.

### 1.2. Our Contribution

In this paper, the novel developed a robot-assisted catheter/guidewire operating system devoted to provide collision detection with haptic cues and take full advantage of the natural manipulation skills of surgeons so as to improve the safe operation of catheterization. In this system, the application of magnetorheological (MR) fluid and high-precision force sensor makes collision detection with haptic cues possible. This method can more readily enable surgeons to distinguish whether the proximal force exceeds or meets the safety threshold of blood vessels. The clamping mechanism based on electromagnetic braking is developed to realize the clamping and relaxation of the catheter. The big contact area and adaptivity of the clamping structure can provide reliable clamping and reduce the clamping injury of the catheter due to the controllability of clamping force [25]. Although the control mode is the same as the scheme employed in the existing master-slave systems, the ergonomic consideration of the input interface and incorporation of natural manipulation skills into the system design is important in ensuring that the system is intuitive to use.

The features of our system are: 1. Do not need to learn new skills to use the robotic system, 2. Be able to take advantage of natural manipulation skills for an experienced surgeon, 3. Adaptively clamp the existing surgical tools and eliminate the need for replacing clamping structures, 4. Be able to operate remotely, avoiding radiation, 5. Proximal force sensing, 6. Quickly collision detection with haptic cues. The design, implementation, and testing to meet the above novelty constitute the main contributions of this paper.

## 2. System Description

**Figure 1:**
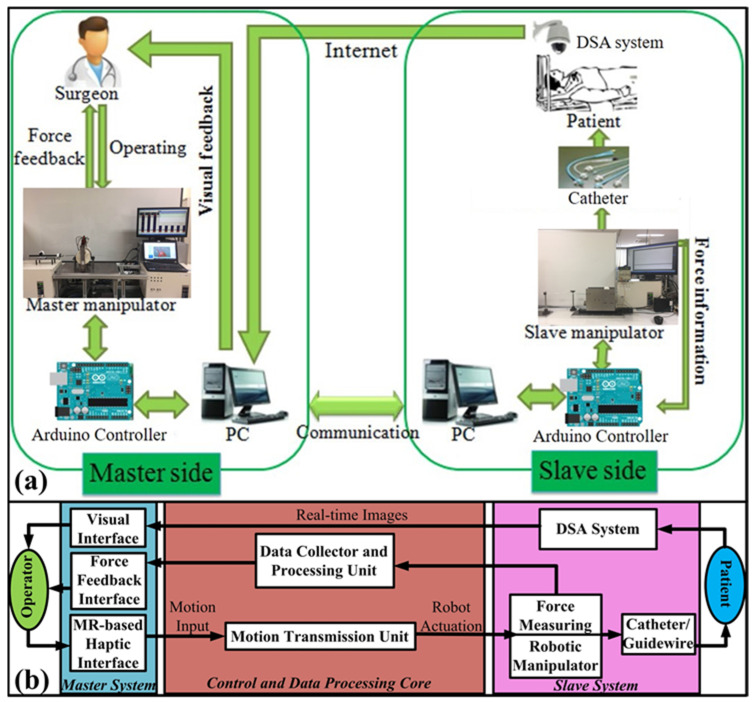
Overview of the endovascular robot-assisted catheter/guidewire operating system. (**a**) Schematic diagram of the proposed robot-assisted catheter/guidewire operating system. (**b**) Signal flow chart of the proposed robot-assisted catheter/guidewire operating system.

Figure 1 illustrates the overview of the endovascular robot-assisted catheter/guidewire operating system, which adopts the master-slave operating system (see Figure 1a). The robot-assisted catheter/guidewire system, a closed-loop architecture, consists of three parts: master subsystem, slave subsystem, and information processing core (see Figure 1b). The master subsystem is composed of a master manipulator incorporating a haptic interface, a force monitor, and a 2D visual monitor. It is used to perform actions similar to those performed during conventional endovascular surgery. The information processing core, including two controllers and the force collector, is used to transmit the motion information to the slave side and feedback the force information to the master side. The slave subsystem composed of a slave manipulator incorporating a force-sensing unit and an imaging navigation system is used to manipulate the surgical tool through the vasculature as commanded by the operator using the master manipulator. The workflow of the system is as follows: A catheter/guidewire manipulated by an operator passes through the master haptic interface, the motion signals are transmitted to the slave manipulator via the motion transmission unit. The slave manipulator with the catheter/guidewire will replicate the same motion in the blood vessel of the patient as the master side. In the meantime, the DSA system on the patient side can provide real-time images to the operator for visual navigation. The high-precision force sensor in the slave side can intuitively display the contact force (tip collision force, friction force, and viscous force) between the catheter and the blood vessel on the PC screen in the form of curves. The force information is also transmitted to the haptic device on the master side. Apart from the force information, accurate localization of the catheter is of importance to realizing endovascular catheterization. Thus, the motion transmission unit feedbacks the position information to the PC screen while transmitting the motion signals so that the operator can quickly acquire the insertion distance of a catheter/guidewire.

### 2.1. Design of the Master Haptic Interface

In recent years, magnetorheological (MR) fluids have been used as the most typical representative of intelligent materials in haptic devices to improve the effectiveness of the human-machine interface because of its high permeability and low hysteresis. Its applications in haptic and force feedback devices have been reported in the literature [26,27,28]. In these studies, haptic systems with MR fluids on the operation side were proposed to acquire tactile sensation for telerobotic surgeries.

**Figure 2:**
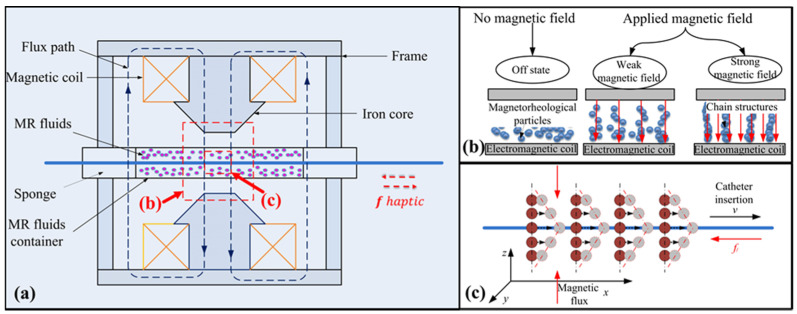
(**a**) Schematic of the MR-based master haptic device. (**b**) The magnetic field characteristic of magnetorheological particles. (**c**) The process of haptic generation in the case of the magnetic field.

Enlightenment based on the above application, in combination with the characteristics of the MR fluids in a magnetic field and the haptic desire of the robot-assisted catheter system, has led our lab to propose a master haptic device based on MR fluids, as shown in Figure 2a. Normally, MR fluids are in a state of fluid-like due to the magnetorheological particles suspended in the nonmagnetic liquid medium. However, the structures under the action of the magnetic field (see Figure 2b) can be transformed from a fluid-like to a solid-like state rapidly and reversibly within milliseconds [29].

The stronger the magnetic field, the greater the strength of the solid-like state is. For this reason, it will generate a resistance force between the MR fluids and the catheter when a rigid rod is inserted into the MR fluids with a magnetic field (see Figure 2c). Based on this, the master haptic interface was developed (see Figure 2a). Since the resistance force can be adjusted by the magnetic field intensity, which is controlled by a current [30]. When a catheter in the slave side encounters the vascular tissue, a current triggered by a transducer signal is sent to the MR haptic device, which creates a slight resistance for the operator associated with the vascular tissue. It is like operating a catheter inside the blood vessel beside a patient in vascular interventional surgery.

**Figure 3:**
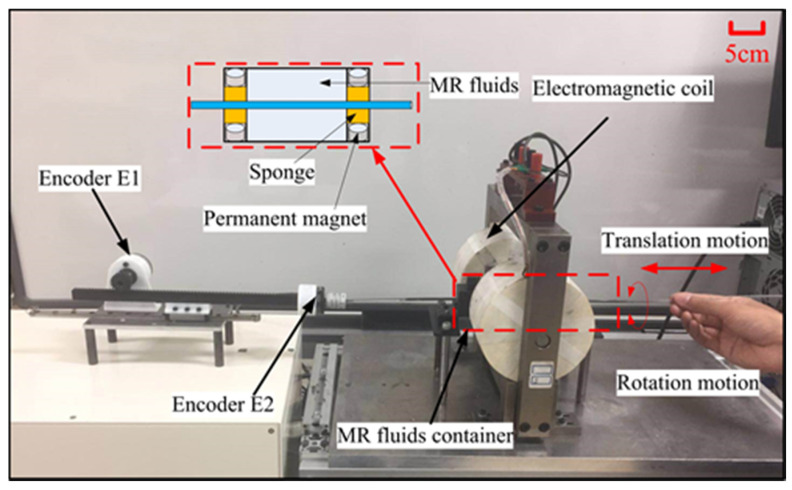
A prototype of the master haptic interface.

As shown in Figure 3, the prototype of the master haptic interface consists of two parts: the haptic interface and the motion transmission unit. The detailed specifications of the haptic interface had been designed by Yin et al. [21]. To prevent the overflow of MR fluids from the small holes of the container and reduce the friction caused by the edge of the holes, the dynamic seal method was proposed. The permanent magnets and sponges were adopted to prevent the overflow of MR fluids (see Figure 3). The magnitude of the permanent magnets is small enough to prevent the MR fluids from flowing out without affecting the friction between the MR fluids and the catheter [21]. The motion transmission unit uses a pinion and rack mechanism mounted on a linear slide. It is coupled to two rotary encoders (MTL, MES020–2000P, Japan), Encoder (E1) and encoder (E2) with 2000 counts/revolution, measures the relative axial displacement of the linear slide and the radial displacement of the rigid rod, respectively. The surgeon’s natural actions, translation, and rotation, during the procedures, are captured by the motion transmission unit. The surgeon uses a rod that passes through the haptic interface attached with the motion transmission unit to perform actions similar to a conventional endovascular procedure. The actions measured by rotary encoders are converted to command signals for the slave manipulator.

### 2.2. Design of the Slave Manipulator

The slave manipulator is used to operate the catheter/guidewire instead of the surgeon’s hands, and it requires a stable clamping and force measurement to realize the insertion, retraction, and rotation of the catheter/guidewire during the catheterization. In addition, various diameters of catheters and guidewires will be used in vascular interventional surgery (VIS). Thus, it is necessary for a clamping structure, which can clamp a catheter/guidewire with different diameters because the replacement of the gripper not only needs to be re-sterilized but also increases the operation time.

**Figure 4:**
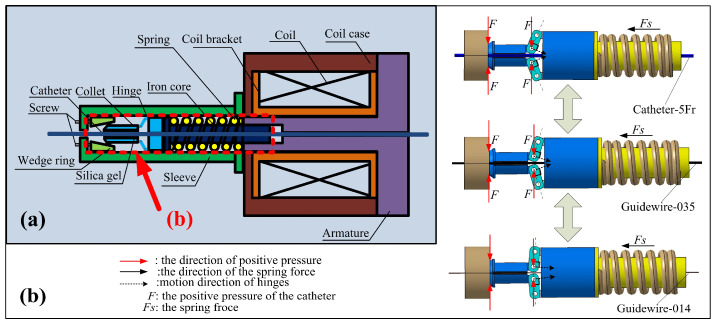
The schematic diagram of the adaptive clamping mechanism. (**a**) An electromagnetic breaking-based adaptive clamping structure. (**b**) The adaptivity of the clamping structure.

Based on these requirements, an electromagnetic breaking-based adaptive clamping structure, shown schematically in Figure 4a, is proposed. When the coil is not energized, the collet will be pressed into the tapered hole of the wedge ring by the spring force (Fs), which can be adjusted by two screws. The collet will generate the positive forces (F) on the catheter/guidewire under the extrusion of the wedge ring and hinges. The resulting static friction forces make the catheter/guidewire and collet into a whole. In addition, the large clamping area and the use of silica gel ensure the stability of the catheter/guidewire clamping due to the increase in static friction force. Figure 4b demonstrates the adaptivity of the clamping structure. The clamping structure will not fail even if the diameter of the clamping object is small.

The two parts of the collet will be maintained close to each other until the object is clamped stably. When the coil is energized, the electromagnetic force generated by the coil can absorb the iron core close to the armature side so that the collet releases the catheter/guidewire because of the disappearance of positive pressure.

**Figure 5:**
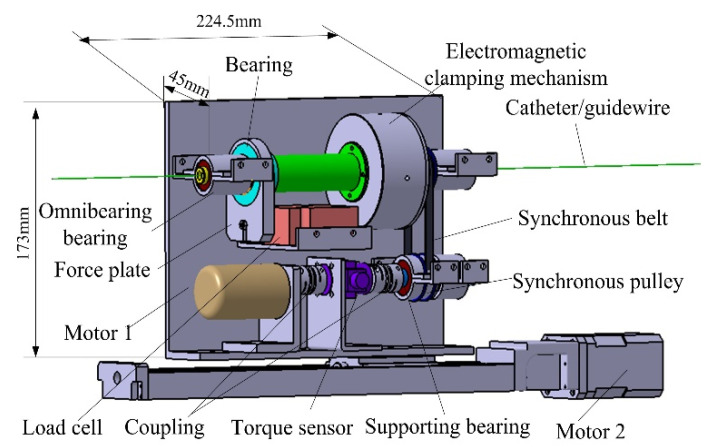
Structure diagram of the slave manipulator.

The slave manipulator, schematically shown in Figure 5. It consists of three parts: the electromagnetic clamping mechanism, force measurement mechanism, and motion driving mechanism [31]. The electromagnetic clamping mechanism has been introduced before (see Figure 4). The force measurement mechanism based on a load cell (resolution: 0.0002) is used to measure the force information between the catheter/guidewire and the blood vessel. The load cell is fixed on the support plate. A force plate fixed on the load cell is linked to the bearing installed on the clamping structure. The existence of the bearing ensures that the rotation action does not interfere with the force measurement. Two omnibearing bearings are used to minimize the influence of friction components on force measurement. A torque sensor placed between the torsional drive motor (motor 1) and the synchronous pulley is used to measure the torsional force. The motion driving mechanism composed of the ball screw and the synchronous belt with two motors achieves imitation of a surgeon’s insertion motion and twisting motion.

## 3. Safety Mechanism

**Figure 6:**
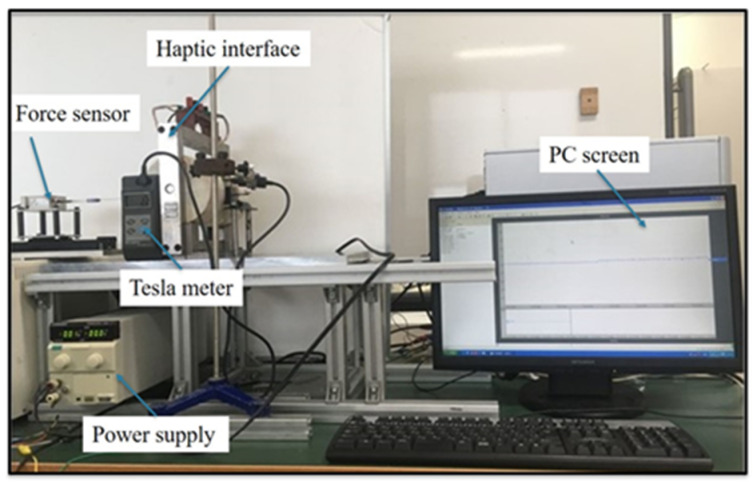
The experimental setup for the haptic force assessment.

To evaluate the effectiveness of collision detection with haptic cues, the dynamic force assessment experiment is performed to achieve the magnitude of the haptic force. The experimental setup is shown in Figure 6. During the experimental process, a rigid rod connected with a load cell (TEAC, TU-UJ, Japan) is actuated by a sliding table to make the insertion motion in the haptic interface. The PC will record and display the generated force information of the haptic interface with and without magnetic fields. Since it is reported that the input velocity does not affect haptic forces, the input velocity is set to 5 mm/s [21]. To maximize the detection effect, the maximum current provided by the power supply is used to obtain the maximum magnetic field intensity, thus achieving the maximum haptic force. The state of MR fluids will be changed from a fluid-like to a solid-like within milliseconds [29].

**Figure 7:**
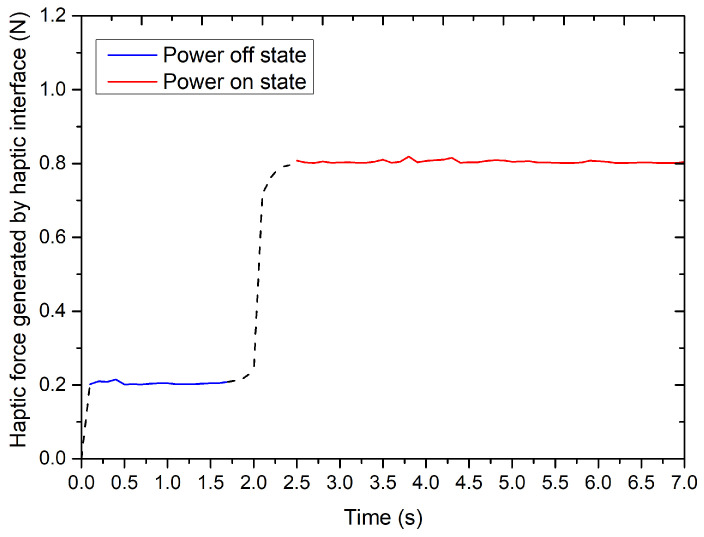
The experimental results for the haptic force assessment.

The experimental results are summarized in Figure 7. From the results, we can see that the dynamic force will quickly change from the initial value to the maximum value when the electromagnetic field is applied. Furthermore, the change of haptic force reaches about 600 mN, which is far greater than 19 mN (human’s finger detection resolution, i.e., just-noticeable difference (JND)) [32]. Therefore, this haptic change can quickly trigger the operator’s stress response and achieve the purpose of collision detection, even for novices.

## 4. Experimental Evaluation

The bilateral teleoperation is defined as an operator operating a robotic system to execute the human’s commands at the local/remote site while receiving position feedback and haptic feedback from the target environment [33]. These two kinds of feedback information can improve the performance of the bilateral teleoperation system [34]. High accuracy and stability of transmission and feedback are the necessary conditions for successful operation. Therefore, stability is an important topic for bilateral teleoperations.

### 4.1. Performance Evaluation of the Bilateral Operation

**Figure 8:**
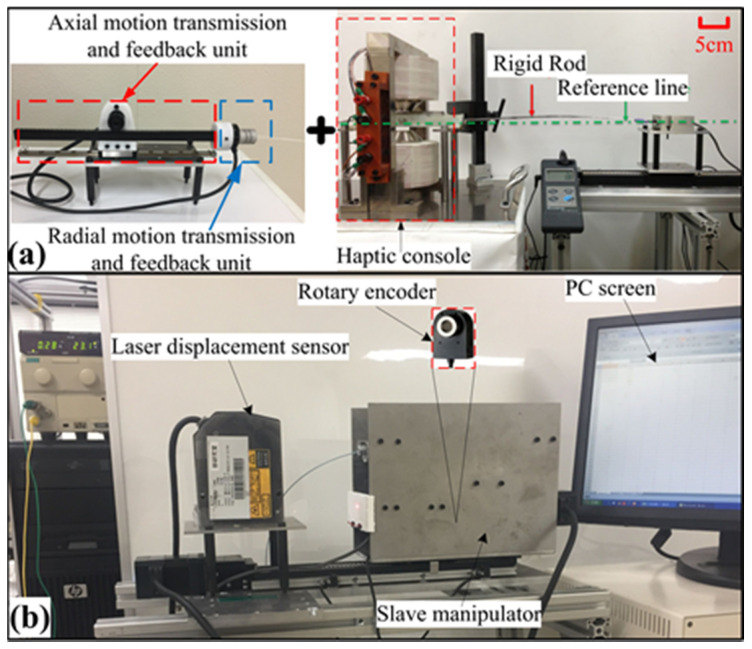
(**a**) The motion input setup on the master side. (**b**) The motion detection setup on the slave side.

The experiment for the bilateral operation, as shown in Figure 8, is carried out. One rotary encoder with pinion and rack serves as an input and feedback device for axial motion (see Figure 8a). The other rotary encoder acts as an input and feedback device for radial motion (see Figure 8a). The positions of the two encoders of the master side were transmitted to two motion controllers after decoding so that the slave robot would follow the axial and radial position of the master. An Arduino Due development board (Smart Projects, Strambino, Italy) incorporates a 32-bit ARM-architecture microcontroller, is used for real-time quadrature decoding and streaming of the position data to the control unit. The control unit communicates with the master motion transmission unit to obtain the desired reference positions and simultaneously measures the positions of the catheter in the slave side by a laser displacement sensor (KEYENCE, LK-500, Japan), and a hollow rotary encoder (MUTOH, UN-2000, Japan) fixed on the drive shaft (see Figure 8b). A control algorithm is used to transmit the motion signals to the motor driver. The accuracy of motion control can be adjusted by controlling the number of pulses transmitted and the frequency of pulse signals. In order to prevent the communication between master and slave has a significant delay, we use the cable connection for master-slave communication. Through a large number of experimental tests, the appropriate control parameters are determined in this algorithm, ultimately realizing the bilateral teleoperation of the system. In addition, the stepper motor (ASM46AA, Oriental Motor CO. LTD.) used in the slave robot is a kind of special motor used for control. Its rotation is operated step by step at a fixed angle (called “step angle”). Its characteristic is that there is no accumulated error.

**Figure 9:**
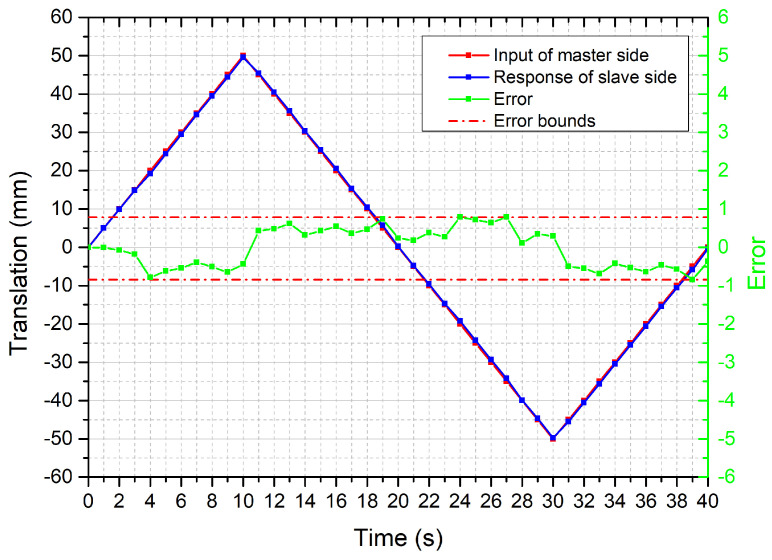
Results for the axial motion transmission.

**Figure 10:**
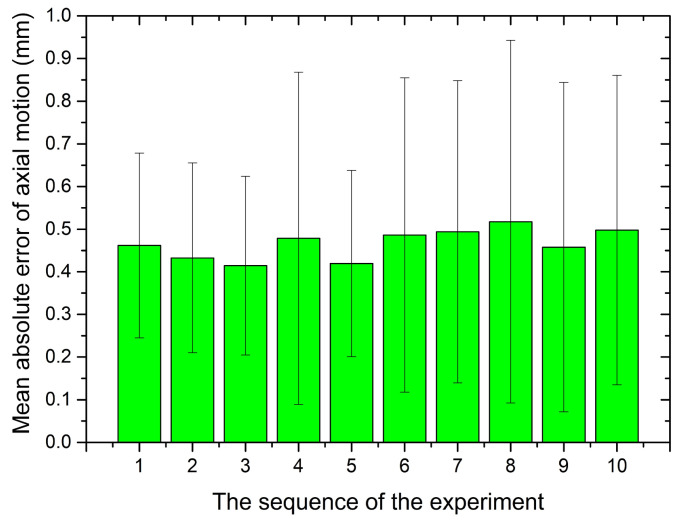
Mean error of ten times experiments for the axial motion.

The bilateral operation experiment for the axial motion was performed. The error in translation, as shown in Figure 9, is bounded between ±0.79 mm. It depicts that the slave robot could precisely follow the trajectory of the master motion input unit that can meet the requirement of the surgery [35]. To verify the stability of the axial motion, the bilateral operation experiment was performed 10 times in the same conditions. The mean absolute error for each trial was summarized in Figure 10.

From the results, we can see that the maximum error is less than 1 mm, but the standard deviations shown in this figure are large, which indicates the errors have a substantial discreteness. The reason may be the deviation of laser detection caused by the tiny vibration produced by the sliding table.

**Figure 11:**
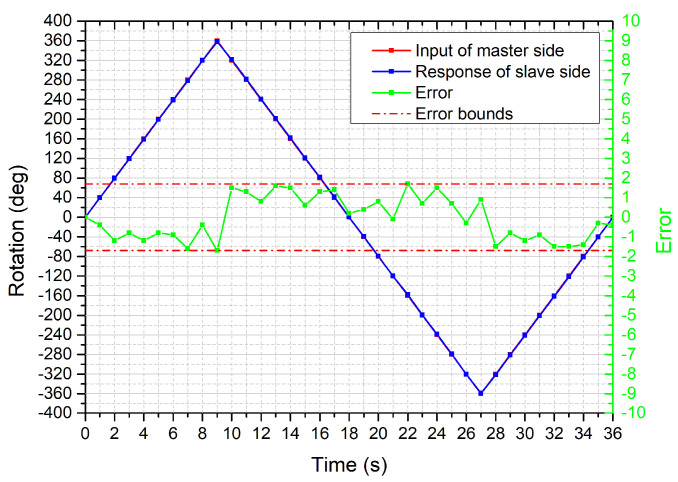
Results for the radial motion transmission.

**Figure 12:**
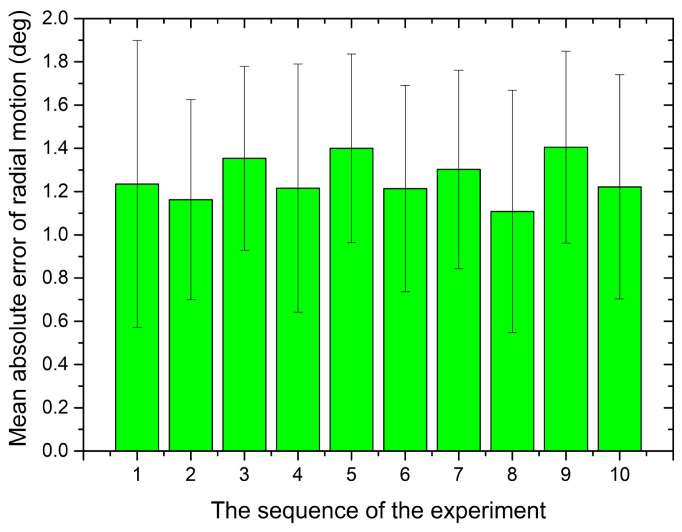
Mean error of ten times experiments for the radial motion.

The bilateral operation experiment to evaluate the radial motion was conducted. The performance of radial motion transmission and the error is shown in Figure 11. The results show that the rotation error is bounded between ±1.7 degrees. After 10 times tests, the mean absolute error for each time was summarized in Figure 12. From the results, the standard deviations relative to their mean values have a significant discreteness. However, it can be acceptable because the absolute error within 2 degrees in this system could not affect the direction adjustment of the catheter/guidewire tip.

### 4.2. Performance Evaluation of the Force-Measuring Unit

In pursuit of telepresence and to increase task performance, the robot-assisted catheter system incorporates force feedback. The most important part of force feedback is the accurate measurement of force information. To prevent the influence of gravity on force measurement, the mechanism is horizontally placed so that the axial direction of the load cell is parallel to the ground, and then combined with omnidirectional bearing as support, the gravity component of the device acting on the axial direction of the force sensor is zero.

#### 4.2.1. Static Characteristic for the Force-Measuring Unit

**Figure 13:**
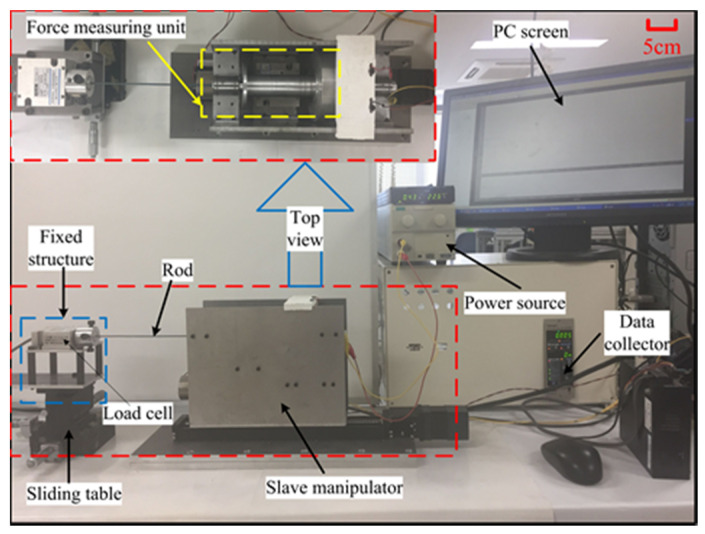
The experimental setup for evaluating the static characteristic.

**Figure 14:**
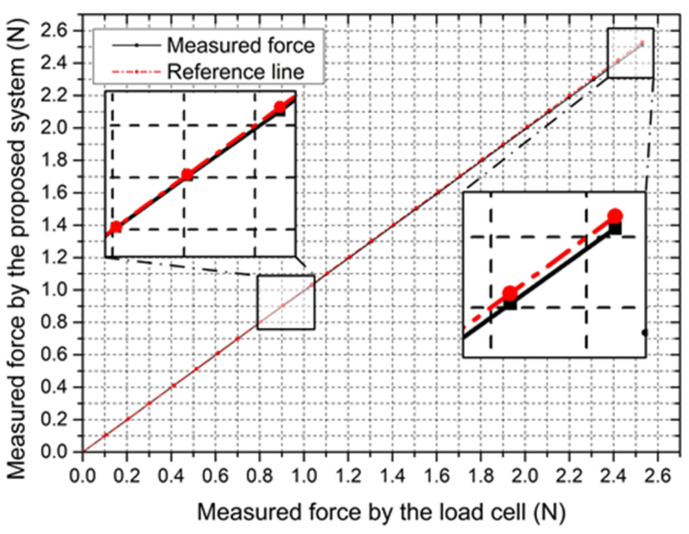
The experimental results for evaluating the static characteristic.

The accuracy is the primary concern for force measurement that requires an accurate force-measuring unit with a fast response. It has an important influence on the safety of surgeries, especially for fragile vessels. To validate the accuracy of the measured force, the step response experiment was performed. As shown in Figure 13, a rod with the silica gel film on the surface was clamped by the slave manipulator, and the other side was firmly connected to the load cell. After limiting the movement of the slave manipulator, the micro-feed of the sliding table increased the force exerted on the rod. The force was increased from 0 N to 2.5 N with a 0.1 N step increase in axial displacement. The force information was collected, respectively, by their own data collectors and displayed on the PC screen. The experiment was performed 10 times for each load, and their mean values are depicted in Figure 14.

From the results, the hysteresis for force measurement was generated, with an average value of 8.89 mN. Hysteresis is very small in the whole load range, particularly at lower load. The hysteresis only slightly increases with the load increase, potentially due to slight loads absorbed by the spring (see Figure 4). When smaller hysteresis is needed, it can be improved by increasing the spring force.

#### 4.2.2. Dynamic Characteristic for the Force-Measuring Unit

**Figure 15:**
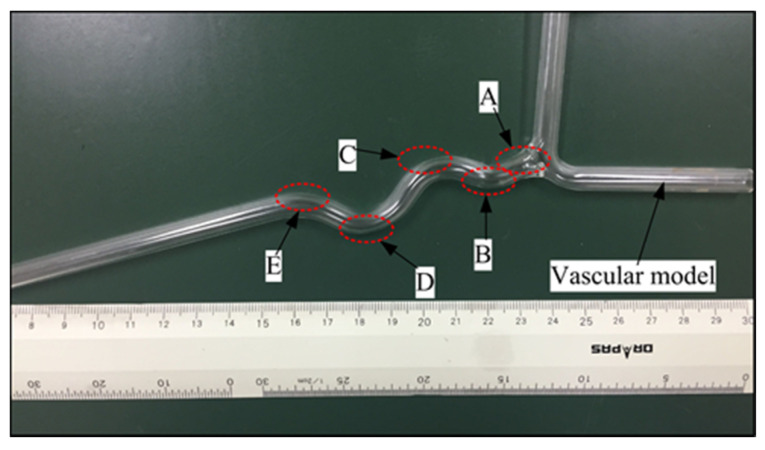
A vascular model for evaluating the dynamic characteristic.

**Figure 16:**
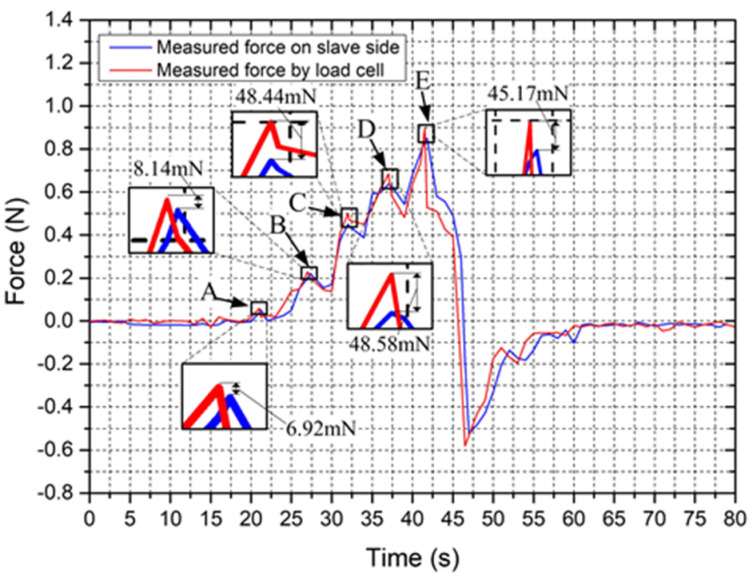
The experimental results for the dynamic characteristic.

For investigating the dynamic characteristic of the force-measuring unit, the proposed slave manipulator is used to track a vascular model shown in Figure 15. In this vascular model, five curved areas are used to compare measurements because the tortuosity of vessels is one of the main failure reasons in endovascular procedures [13]. The experimental setup is displayed in Figure 13. A catheter-5Fr clamped by the slave manipulator was controlled to perform the insertion and retraction motion in the vascular model at 3.5 mm/s. When the catheter-5 Fr comes into contact with the vascular model, the force generated from the slave side is transmitted to the data acquisition unit. Next, the catheter-5Fr is directly connected with the load cell to perform the same insertion and retraction motion. The experimental process is as follows: the catheter passes through the A, B, C, D, E point of the vascular model in sequence under the action of the slave platform. When the tip of the catheter reaches the E point, the catheter retracts until it completely exits the vascular model. The results of force tracking were displayed in Figure 16. From the results, we can see that, when the catheter tip passed through the curved regions (areas with risk of perforation), the force measured from the slave side in the insertion process was slightly smaller than the actual force with a maximum error of 48.58 mN, which was acceptable in the actual surgery [36]. The maximum errors were found in the two regions (C and D) with the greatest degree of curvature. Perhaps the reason for this case is due to small loads absorbed by the spring and the difference in catheter deformation, which can cause different friction forces of the catheter. However, even with the technical difficulty, the usability of force feedback increased by having the force information available.

## 5. In Vitro Evaluation

In vascular interventional surgery, the catheter must pass through several curved areas continuously to reach the lesion target. To prevent any bending areas from being punctured, it is also necessary for tip collision cues to be transmitted in haptic cues except force feedback.

### 5.1. Performance Evaluation

**Figure 17:**
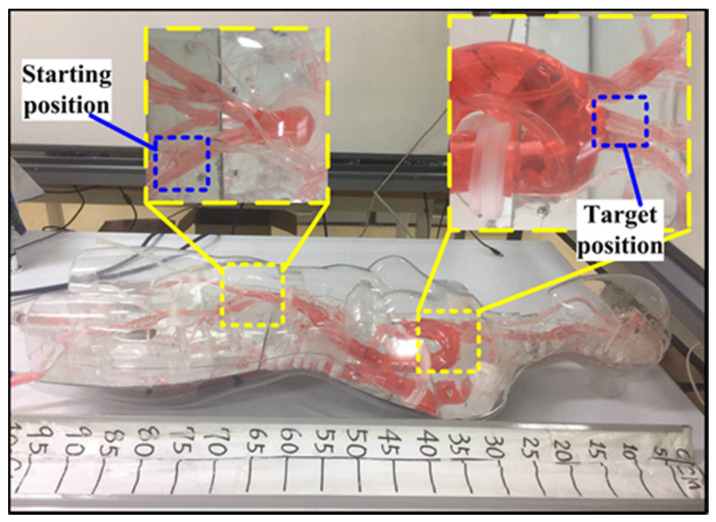
The EVE model for the evaluation experiment.

A simple insertion experiment was carried out to test the collision detection capability of the proposed system. The experimental object is the human body model called Endo Vascular Evaluator (EVE: FAIN-Biomedical, Nagoya, Japan), as shown in Figure 17. The starting position and the target position pressure are set to 120/80 mmHg. The actual task that the user(non-medical) has to complete is that the catheter is operated from starting position to the target position without damaging the blood vessels. The experimental conditions were considered as two modes. Each mode contained visual feedback by an Internet protocol (IP) camera and traditional force feedback. Mode1: haptic feedback (HF) means collision detection with haptic cues; Mode2: no haptic feedback (NHF) means just intuitive force feedback.

**Figure 18:**
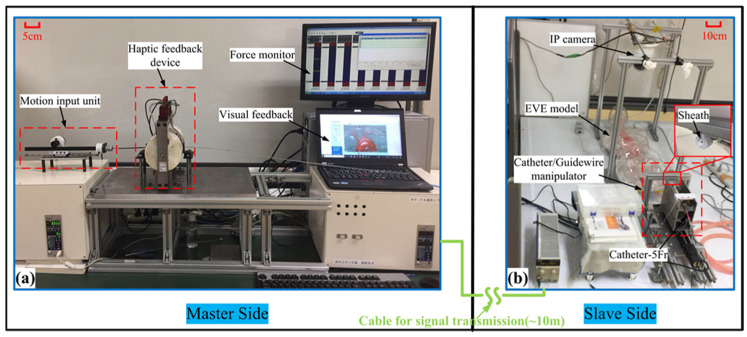
Experimental setup of collision detection performance for the presented system. (**a**) The master side of the presented system. (**b**) The slave side of the presented system.

The experimental setup is shown in Figure 18. The master side was operated inside the control room. The force and visual monitor were placed at the master side (see Figure 18a). The slave side was placed nearby the EVE model inside the operation room (see Figure 18b). According to previous studies [30,37] and practical tests, the maximum measuring force of the catheter from the starting position to the target position for the EVE model is about 1 N without severe tip collision. Thus, the safety threshold is set to 1 N. The safety threshold of real human blood vessels should be comprehensively judged by experienced surgeons according to the actual blood vessel status and blood viscosity of each patient. Because the individual constitution is not the same, the elasticity of blood vessels is not the same. Blood vessels with age, disease, and other reasons will become weak elasticity, vascular sclerosis. For mode 1, when the measured force exceeds the safety threshold value, the shear force will be generated on the catheter in the haptic interface to increase resistance sensation, thus realizing collision detection obviously.

**Figure 19:**
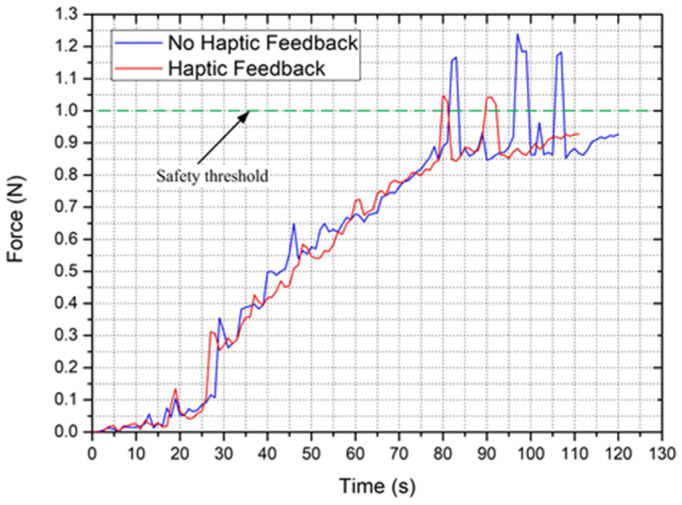
Experimental results of collision detection performance for the presented system.

The experimental results are shown in Figure 19. In mode 2-NHF, the maximum force information (tip collision occurs) measured by the system is much greater than the setting threshold value, the exceeding value is significantly greater than 0.12 N, and the risk of vascular perforation has emerged [36,38].

In mode 1, the maximum force information obtained by the system is much smaller than that in mode 2. Although it also exceeds the setting threshold, the exceeding value is less than 0.12 N, and the safety is greatly improved. In addition, the operation time is also greatly improved. In addition, the operation time is also significantly reduced.

The reason is that there is a reaction time from the tip collision to adjust the position of the catheter. In such a short moment, a slight forward displacement of the catheter can aggravate the collision. This situation is very dependent on the technical and psychological endurance of surgeons. However, when a collision is a feedback to a surgeon in haptic cues, the fastest response will be performed. In addition, the haptic device in this system not only prompts the surgeon to be aware of collisions but also produces a sudden viscous resistance at that moment for the operation rod at the master side that prevents the catheter from moving forward. This reduces the continuing increase in the collision force and increases the margin of safety of the operation. In addition, haptic cues can reduce the time required to monitor the force and the surgeon’s psychological pressure caused by the untimely response of the collision. The system permits the surgeon to operate the catheter more attentively and smoothly, thus reducing the operation time.

### 5.2. Validation Trials

To further demonstrate the performance of the proposed system in collision detection with haptic cues, trials were performed with 10 different subjects (non-medical) who had no prior experience with either setup (mode 1 and mode 2) in the same experimental conditions. The advantage of this experimental design is that it provides a clear, unbiased performance comparison because it eliminates any learning bias. The experimental task was the same as performed above. Each subject performed 10 trials with each mode for statistics purposes. The following two important metrics were considered in this experiment: (1) the average maximum force of the task and (2) the average elapsed time of the task accomplishment.

**Figure 20:**
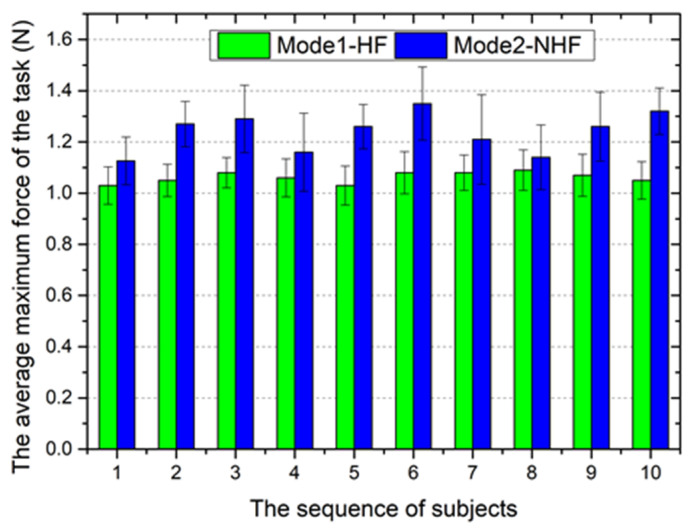
The average maximum force of the task accomplishment.

Based on the results summarized in Figure 20, for each subject, the average maximum force of the task with collision detection is smaller than that without collision detection. Especially in cerebrovascular surgery, the vascular wall is easy to pierce due to its fragility. Therefore, the collision detection with haptic cues of the robotic catheter system played an important role in tissue protection.

**Figure 21:**
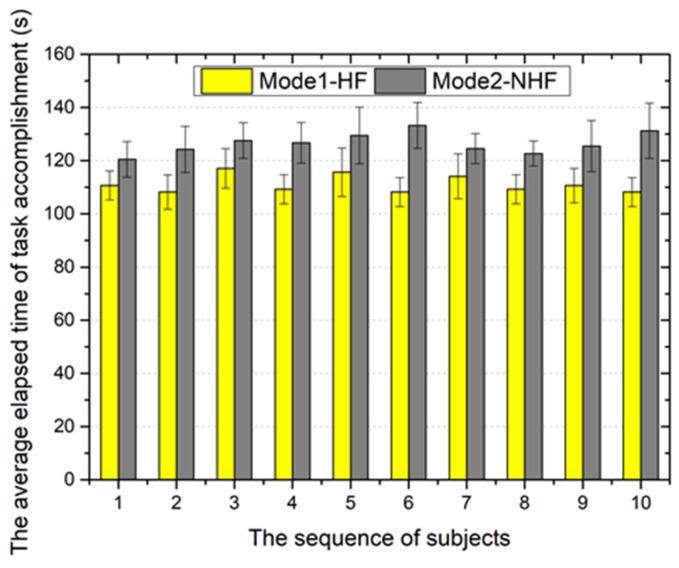
The average elapsed time of task accomplishment.

Figure 21 shows the average elapsed time of task accomplishment for all participating subjects. The statistical results indicate that the average elapsed time of mode 1 is shorter than that of mode 2. The primary reason for this reduction has been analyzed above. The reduction in operating time can reduce the time of radiation exposure for the patient, thereby reducing the radiation damage to the human body.

In general, the proposed robotic catheter system with collision detection with haptic cues can reduce the operating time and quickly alert the surgeon to the occurrence of collisions. This makes the surgeon respond to adjust a catheter/guidewire promptly, thus reducing the risk of vascular perforation caused by collisions and improving the safety of the operation.

## 6. Conclusions

In this paper, a robot-assisted catheter/guidewire system is successfully developed and demonstrated to improve the safe operation of catheterization. The master-slave scheme of the robotic catheter system is designed to take full advantage of natural manipulation skills for a surgeon. The bilateral teleoperation was conducted to evaluate the transmission accuracy in the axial and radial directions. The accuracy of the measured force has an important influence on the safety of surgeries. The experiments of the static characteristic and dynamic characteristic for the proposed force-measuring unit were performed to validate the capacity of force measurement. Besides the synchronization of motion and adequate force feedback during the endovascular catheterization, it is essential to provide collision detection to avoid further collision trauma and vessel puncture effectively. The application of the haptic interface based on magnetorheological (MR) fluid and high-precision force sensor makes the collision cues feedback to the surgeon in haptic cues. The performance evaluation experiments in vitro of the collision detection for the endovascular robot-assisted system have been carried out. Based on the results, the proposed system has made a great contribution to reducing the maximum contact force and average elapsed time for completing the task, thus effectively improving the security of operation during the endovascular procedures.
